# Cleft lip/palate and educational attainment: cause, consequence or correlation? A Mendelian randomization study

**DOI:** 10.1093/ije/dyaa047

**Published:** 2020-05-06

**Authors:** Christina Dardani, Laurence J Howe, Nandita Mukhopadhyay, Evie Stergiakouli, Yvonne Wren, Kerry Humphries, Amy Davies, Karen Ho, Seth M Weinberg, Mary L Marazita, Elisabeth Mangold, Kerstin U Ludwig, Caroline L Relton, George Davey Smith, Sarah J Lewis, Jonathan Sandy, Neil M Davies, Gemma C Sharp

**Affiliations:** d1 Centre for Academic Mental Health, Population Health Sciences, Bristol Medical School, University of Bristol, Bristol, UK; d2 MRC Integrative Epidemiology Unit, Population Health Sciences, Bristol Medical School, University of Bristol, Bristol, UK; d3 Institute of Cardiovascular Science, University College London, London, UK; d4 Centre for Craniofacial and Dental Genetics, Department of Oral Biology, University of Pittsburgh, Pittsburgh, PA, USA; d5 The Cleft Collective, University of Bristol, Bristol, UK; d6 Bristol Speech and Language Therapy Research Unit, North Bristol NHS Trust, Bristol, UK; d7 Bristol Bioresource Laboratories, Population Health Sciences, Bristol Medical School, University of Bristol, Bristol, UK; d8 Institute of Human Genetics, University of Bonn, Bonn, Germany; d9 Department of Genomics, Life and Brain Center, University of Bonn, Bonn, Germany; d10 Dean of the Faculty of Health Sciences, University of Bristol, Bristol, UK

**Keywords:** Non-syndromic cleft, educational attainment, Mendelian randomization, IQ, orofacial cleft, cleft lip and palate, intelligence

## Abstract

**Background:**

Previous studies have found that children born with a non-syndromic orofacial cleft have lower-than-average educational attainment. Differences could be due to a genetic predisposition to low intelligence and academic performance, factors arising due to the cleft phenotype (such as social stigmatization, impaired speech/language development) or confounding by the prenatal environment. A clearer understanding of this mechanism will inform interventions to improve educational attainment in individuals born with a cleft, which could substantially improve their quality of life. We assessed evidence for the hypothesis that common variant genetic liability to non-syndromic cleft lip with or without cleft palate (nsCL/P) influences educational attainment.

**Methods:**

We performed a genome-wide association study (GWAS) meta-analysis of nsCL/P with 1692 nsCL/P cases and 4259 parental and unrelated controls. Using GWAS summary statistics, we performed Linkage Disequilibrium (LD)-score regression to estimate the genetic correlation between nsCL/P, educational attainment (GWAS *n* = 766 345) and intelligence (GWAS *n* = 257 828). We used two-sample Mendelian randomization to evaluate the causal effects of genetic liability to nsCL/P on educational attainment and intelligence.

**Results:**

There was limited evidence for shared genetic aetiology or causal relationships between nsCL/P and educational attainment [genetic correlation (rg) −0.05, 95% confidence interval (CI) −0.12 to 0.01, *P* 0.13; MR estimate (βMR) −0.002, 95% CI −0.009 to 0.006, *P* 0.679) or intelligence (rg −0.04, 95% CI −0.13 to 0.04, *P* 0.34; βMR −0.009, 95% CI −0.02 to 0.002, *P* 0.11).

**Conclusions:**

Common variants are unlikely to predispose individuals born with nsCL/P to low educational attainment or intelligence. This is an important first step towards understanding the aetiology of low educational attainment in this group.

**Key Messages**

- Some previous studies have found that children born with a cleft lip with or without cleft palate have lower-than-average educational attainment, even in the absence of other conditions or known syndromes.
- It has been suggested that these differences could be due to a genetic predisposition for low intelligence caused by undiagnosed congenital differences in brain structure or function.
- Alternatively, these differences might be explained by downstream factors related to having a cleft, such as social stigmatization, impaired speech and language development or confounding factors such as family socio-economic position or parental health behaviours (e.g. smoking or drinking alcohol).
- This study suggests that common genetic variants are unlikely to predispose individuals born with a non-syndromic cleft lip with or without cleft palate to low educational attainment or intelligence.
- This information could have an important impact on family counselling and coping strategies, and on the self-concept and public perception of people born with a cleft.
- Our findings also encourage further research into possible explanations for observed associations between non-syndromic orofacial clefts and lower educational attainment, in particular the possible contribution of downstream factors related to having a cleft.
- In the current absence of any targeted educational interventions or supportive policies for individuals born with a cleft, such research will be an important step towards improving educational outcomes in this group.

## Introduction

Worldwide, orofacial clefts affect around one in 600–700 live births.^1^ Although these structural anomalies can be surgically repaired (in regions where access to care is available), the condition remains associated with multiple adverse outcomes that can persist into adulthood, including impaired speech, appearance concerns and suboptimal psychological wellbeing.^2^^,^^3^

Some evidence suggests that children born with orofacial clefts are at higher risk of low educational attainment, even when there are no other major birth defects or known syndromes. Small studies dating back to the 1950s have reported lower mean IQ scores, higher rates of learning difficulties and lower educational attainment in cases compared with controls or general-population averages.^4–9^ Although some of these early studies were susceptible to selection and outcome measurement biases, some of their findings have been corroborated by more recent, population-based studies. In a data-linkage study in Atlanta, children with isolated clefts were two times more likely to use special-education services than children with no major birth defects, whereas the broader group of children with any orofacial cleft (i.e. isolated or occurring with another condition) were three times more likely to use these services.^10^ A Swedish population-based registry study showed that children with cleft lip and palate were less likely to receive high grades compared with over 1.2 million controls.^11^ Children with cleft palate only were even less likely to receive high grades. Similarly, studies based on registry data in Iowa showed that children with a non-syndromic cleft were approximately half a grade level behind their classmates,^12^ with persistent low achievement trajectories^13^ observed predominantly in children with cleft palate, but also in children with isolated cleft lip with or without cleft palate. Interestingly however, achievement scores were similar between affected children and their unaffected siblings.^14^ In the most recent population-based study, 2802 5-year-old children born with a non-syndromic cleft in England had lower average academic achievement across all learning domains compared with national averages, with clefts involving the palate accounting for the biggest differences.^15^ Overall, the existing evidence suggests that, although the academic achievement of children with cleft lip with or without palate is less affected than children with cleft palate only, they are still at risk of worse academic outcomes compared with their peers.

Low educational attainment can have a long-lasting adverse impact on vocational, social, mental and physical health outcomes.^16^ Interventions and policies to improve educational attainment in individuals born with a cleft could have wide-ranging knock-on effects on their quality of life. However, it is currently unclear what the targets of such interventions should be, and indeed whether these targets are even modifiable by intervention.

**Figure 1.**
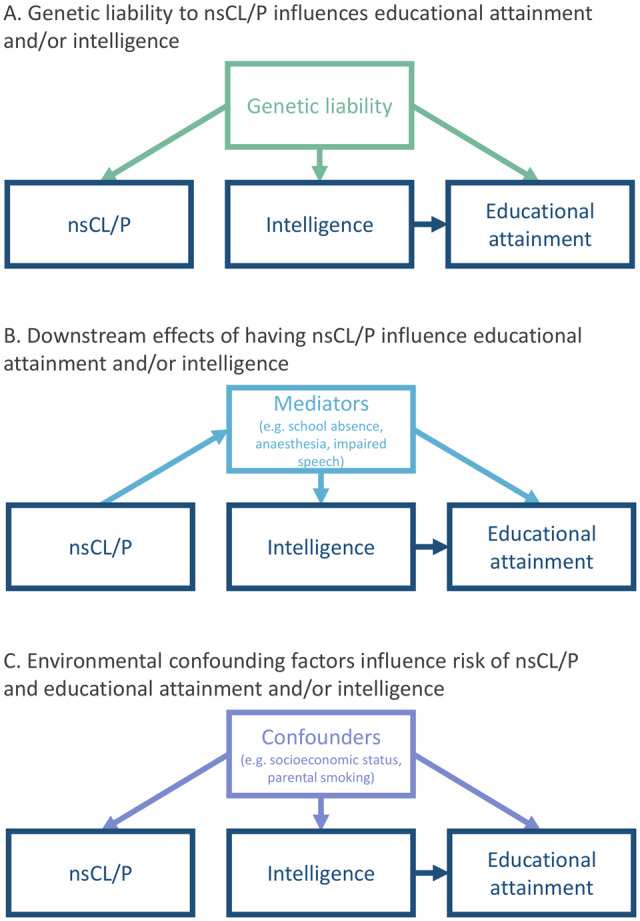
Potential explanations for observed associations between non-syndromic cleft lip with/without palate (nsCL/P) and lower educational attainment. In this study, we use genetic variants to assess whether individuals born with nsCL/P are genetically predisposed to low educational attainment (Explanation A).

Therefore, we need to understand why individuals born with isolated, non-syndromic orofacial clefts [non-syndromic cleft lip with or without palate (nsCL/P)] might have a higher risk of lower educational attainment. Three potential explanations for these associations are:

an underlying genetic liability to develop a cleft also influences intelligence and academic ability,^17^ potentially via subtle undiagnosed congenital differences in brain structure or function^18^^,^^19^; such effects could be caused by common or rare genetic variants;

factors related to being born with the nsCL/P phenotype influence educational attainment; such factors include time spent under anaesthesia,^20^ a high number of school absences due to healthcare appointments, social stigmatization (e.g. due to teasing by peers^21^ or perceptions and expectations of teachers^22^) lower self-esteem, or impaired speech,^23^ or delayed language development^24^;

environmental confounding by factors such as parental health behaviours or family socio-economic status^14^ (Figure 1).

In this study, we assessed evidence for the hypothesis that genetic liability to nsCL/P, as captured by common genetic variation, influences educational attainment (Explanation A). Identified common genetic variants explain between 30% and 40% of the heritability of nsCL/P. Every individual can be assumed to have an underlying common variant genetic liability to nsCL/P, which is normally distributed across the whole population.^25^ Assuming a threshold model of inheritance (as previous evidence supports^25^), genetic liability above a threshold will lead to the phenotypic expression of nsCL/P, whereas increased liability below the threshold could lead to the expression of some aspects relating to the trait. For example, in previous work, we have shown that common variant genetic liability to nsCL/P influences decreased philtrum width in individuals without a cleft,^17^^,^^25^ so a similar relationship might exist for educational attainment and intelligence. In this study, we combined genome-wide association study (GWAS) summary statistics from several previous studies in a meta-analysis including 1692 nsCL/P cases and 4259 parental and unrelated controls of European descent (the database of Genotypes and Phenotypes, dbGAP, accession numbers: phs000094.v1.p1; phs000774.v2.p1; and Bonn-II-study). We used Linkage Disequilibrium (LD)-score regression^26^ to estimate the genetic correlation between liability to nsCL/P, educational attainment and intelligence. We then performed bidirectional two-sample Mendelian randomization (MR)^27^^,^^28^ to investigate any causal effect of genetic liability to nsCL/P on these two traits. A clearer understanding of this mechanism will help tailor interventions to improve educational attainment in individuals born with nsCL/P.

## Methods

We used LD-score regression and MR to assess whether the association of nsCL/P and low educational attainment relates to genetic predisposition to low educational attainment or low intelligence. This analysis used summary statistics from published GWAS.

### Samples (GWAS summary statistics)

#### GWAS meta-analysis of nsCL/P

For nsCL/P, we performed a meta-analysis of GWAS summary statistics from three sources: the Bonn-II study,^29^ the International Cleft Consortium (ICC; dbGaP Study Accession phs000094.v1.p1) and the Pittsburgh Orofacial Cleft (POFC) Study run out of the University of Pittsburgh (dbGaP Study Accession phs000774.v2.p1).

Information on the generation of the GWAS statistics from the Bonn-II study and the ICC can be found in Howe *et al*.^25^ This paper also shows that meta-analysing the Bonn-II and the ICC data (1215 nsCL/P cases and 2772 parental and unrelated controls; total *n* = 3987) produces summary statistics, which are comparable to those generated by a previous meta-analysis published by Ludwig *et al*.,^30^ which used a similar approach in a sample of 666 European and European American trios and 795 Asian trios, combined with 399 cases and 1318 controls of European ancestry. Summary statistics from Ludwig *et al*. were not publicly available.

Subjects of European descent were selected from the POFC study excluding samples that overlapped with the ICC. POFC ethics approval was obtained from the University of Pittsburgh IRB, FWA00006790. We conducted genome-wide association using the transmission disequilibrium test in 978 nsCL/P family trios and case–control association in 151 cases and 835 unrelated controls. Association outcomes from the two POFC GWAS were meta-analysed along with the Bonn-II and ICC study using the methods described in the Supplementary data available at *IJE* online.

For all GWAS, non-syndromic cases were ascertained based on detailed clinical assessment, in order to identify any co-morbid developmental and congenital abnormalities that could suggest a syndrome. The sample was restricted to cases with nsCL/P. Cases with isolated palate or ‘unknown cleft’ were excluded.

We conducted a meta-analysis of summary statistics from all three sources (Bonn-II, ICC, POFC) using METAL^31^ and a previously described protocol for combining TDT and case–control studies.^32^ In total, we meta-analysed GWAS summary data on 1692 nsCL/P cases and 4259 parental and unrelated controls.

#### Educational attainment

For educational attainment, we used publicly available GWAS summary statistics published by Lee *et al*.^33^ (downloaded from https://www.thessgac.org/data), with a total sample size of 766 345 individuals. This was the total sample size available, excluding data from 23andMe due to restrictions on data sharing. Educational attainment was defined by mapping qualifications onto the International Standard of Classification of Education and was converted into years of education (in adults). This definition of educational attainment is strongly associated with other measures of educational attainment, including achieved grades and test scores.^34^

#### Intelligence

For intelligence, we used publicly available GWAS summary statistics published by Lee *et al*.^33^ (downloaded from https://www.thessgac.org/data), with a total sample size of 257 828 individuals. These summary statistics were generated by a meta-analysis of independent GWAS from UK Biobank and the COGENT consortium.^35^ UK Biobank measured intelligence using a standardized score from a verbal–numerical reasoning test, designed as a measure of fluid intelligence. COGENT used a measure of intelligence based on performance on at least three neuropsychological tests or at least two IQ-test subscales. More information on phenotype definitions and generation of these GWAS summary statistics is available in Lee *et al*.^33^

### LD-score regression

We used LD-score regression to estimate the genetic correlation between liability to nsCL/P and both educational attainment and intelligence. LD-score regression uses patterns of LD among genetic variants to estimate the extent of shared genetic aetiology among polygenic traits, accounting for cryptic relatedness and stratification.^26^ We estimated genetic correlations using the suggested protocol for the LD-score regression software for Python,^26^ with pre-computed LD scores from the 1000 Genomes project,^36^ available from the Broad Institute (https://data.broadinstitute.org/alkesgroup/LDSCORE/). In the regression analyses, we used an unconstrained intercept to account for (unknown, but unlikely) sample overlap.

LD-score regression can provide unreliable estimates with small GWAS (such as the nsCL/P GWAS we are using). We assessed the reliability of our estimates using the conditions set out by developers of the approach, namely that the heritability (H^2^) Z score is at least 1.5 (optimal >4), the mean chi square is >1.02 and the intercept estimated from the single nucleotide polymorphism (SNP) heritability analysis is between 0.9 and 1.1.^37^ We also conducted a positive control analysis with philtrum width (*n* = 6136)—a trait known to have shared genetic aetiology with nsCL/P.^25^

### Bidirectional two-sample MR

#### The causal effect of genetic liability to nsCL/P on educational attainment or intelligence

We applied two-sample summary statistic MR to assess whether genetic liability to nsCL/P influences educational attainment and intelligence. This approach enables estimation of causal effects from GWAS summary statistics. MR uses genetic-variant SNPs as proxies for the exposure that are not subject to confounding and reverse causation.^28^ The three main assumptions of MR are that (i) SNPs are reliably associated with the exposure; (ii) there are no confounders of the SNP-outcome association; and (iii) the SNPs do not directly influence the outcome via a pathway independently of the exposure. The effect of the exposure on the outcome is calculated as the ratio of the SNP effect on the outcome by the effect of the SNP on the exposure. We conducted our two-sample MR analyses using the two-sample MR package for R.^38^

We selected all genome-wide significant (*P*val ≤ 5 × 10^–8^) and independent (*r*^2^ < 0.01; kb window = 10.000) SNPs from our GWAS meta-analysis of nsCL/P. By including only instruments below the genome-wide significance threshold, we reduce the likelihood of including SNPs with spurious horizontally pleiotropic effects (which would violate the third main assumption of MR).

As a sensitivity analysis, we also performed MR using 12 SNPs found to be strongly associated with nsCL/P in a previous nsCL/P GWAS meta-analysis published by Ludwig *et al*.,^30^ conducted on a mixture of Europeans and Asians. Effect sizes and standard errors for these 12 SNPs were extracted from the GWAS meta-analysis conducted in the present study, in order to satisfy the MR requirement for exposure and outcome samples from the same ancestry (given that the educational attainment and intelligence GWASs were conducted exclusively in Europeans).

Details on the SNPs used in the primary as well as sensitivity analyses can be found in Supplementary Tables 1 and 5, available as Supplementary data at *IJE* online.

SNP-outcome effect estimates and standard errors were extracted from the educational attainment and intelligence GWAS summary statistics described above.

Our primary analysis uses the inverse variance weighted (IVW) method. This method calculates the causal effect of genetic liability to nsCL/P (the exposure) on education/intelligence (the outcome) as the ratio of the SNP-outcome effect to the SNP-nsCL/P effect, whereby the ratio derived from each SNP is weighted to its relative precision. We assessed the strength of the instruments by estimating the mean F-statistic. As a rule of thumb, if the mean F > 10, then the IVW is unlikely to suffer from weak instrument bias. We then conducted a series of sensitivity analyses to test the validity of the findings derived by the IVW approach. Specifically, we tested the consistency of our results to those obtained by MR Egger,^39^ weighted-median^40^ and the weighted-mode estimators.^41^ MR Egger estimates the causal effect of the exposure on the outcome allowing for some types of pleiotropic effects. The weighted-median approach provides a causal-effect estimate assuming that at least 50% of the SNPs in the analysis are valid instruments (i.e. the SNPs’ effect on the outcome is unconfounded and entirely mediated via the exposure). The weighted-mode approach provides a causal estimate of the exposure on the outcome assuming the most common effect estimates come from SNPs that are valid instruments.

MR estimates and confidence intervals are expressed as a one-unit increase in the log odds of genetic liability to nsCL/P on standard deviations of years of education/IQ. To aid interpretation, we converted MR estimates into a scale describing the effect of a doubling in the genetic liability to nsCL/P on years of education or IQ points. To do this, we multiplied the original results by the standard deviation for the respective outcome (years of education SD = 4.2, IQ SD = 15) as published by Lee *et al*.^33^ We then multiplied these figures by ln2 to calculate the effect of a doubling of liability to nsCL/P.

### The causal effect of educational attainment or intelligence on genetic liability to nsCL/P

We also applied two-sample MR in the reverse direction, to assess the causal effects of educational attainment and intelligence on offspring liability to nsCL/P. Since clefts form in the first 10 weeks of embryonic development, any effect of education or intelligence will reflect parental effects—due to either the passive transmission of parental genetics or phenotypic expression of parental genetics that influences liability to nsCL/P in the offspring.^42^ That is, nsCL/P cannot arise due to the child’s own education or intelligence, but parental genetic predisposition to low educational attainment or intelligence may influence the early prenatal environment to increase the risk of nsCL/P.^43^ Any parental effect can be inferred as being due to shared (50% from each parent) parent–offspring genetics.^42^

Of the 7 618 724 SNPs in the GWAS of nsCL/P, 6 693 634 were overlapping with the GWAS of educational attainment and 6 693 658 with the GWAS of intelligence. Of these overlapping SNPs, 30 349 and 13 621 had an effect allele frequency ≥0.01 and a *P*-value <5 × 10^–8^ for the association with educational attainment or intelligence, respectively. After LD clumping, 477 approximately independent SNPs (*r*^2^ = 0.01, with a 10 000 kb window) were selected as instruments for educational attainment and 181 for intelligence (Supplementary Tables 8 and 10 and Supplementary Figures 3 and 4, available as Supplementary data at *IJE* online.). We conducted IVW MR combining the SNP-educational attainment/intelligence and SNP-nsCL/P coefficients to give causal-effect estimates of (parental) educational attainment and (parental) intelligence on (offspring) liability to nsCL/P, followed by sensitivity analyses.

MR estimates and confidence intervals are expressed as odds ratios for the effect of a one standard deviation unit increase in education/IQ on the odds of developing nsCL/P. To aid interpretation, we converted MR estimates into odds ratios for the effect of an extra year of education/an extra IQ point on the odds of developing nsCL/P. To do this, we converted to log odds and divided by the standard deviation for the respective traits (years of education SD = 4.2, IQ SD = 15) as published by Lee *et al*.^33^ We then exponentiated these figures to convert to odds ratios.

### Data and code availability

All the summary statistics required to conduct the MR analyses described in this paper are provided in the Supplementary data available at *IJE* online. The code for the analyses can be found in a GitHub repository: https://github.com/ChristinaDni/nsCleftLipPalate_EducationalAttainment.

### Role of the funding sources

No funding body has influenced data collection, analysis or its interpretation.

## Results

### GWAS meta-analysis of nsCL/P

The nsCL/P GWAS meta-analysis (1692 cases, 4259 parental and unrelated controls) summary statistics were clumped in Plink using the 1000genomes phase3 European ancestry reference panel. We identified nine genome-wide significant (*P* < 5 × 10^–8^) and independent (*r*^2^ < 0.01; kb = 10.000) SNPs. Manhattan and QQ plots of the GWAS meta-analysis *p*-values are shown in Supplementary Figures 1 and 2, available as Supplementary data at *IJE* online.

### Genetic correlation

Using LD-score regression, we found little evidence of a substantial genetic correlation between liability to nsCL/P and educational attainment [genetic correlation coefficient (rg) −0.05, 95% confidence interval (CI) −0.12 to 0.01, *P* 0.13] or intelligence (rg −0.04, 95% CI −0.13 to 0.04, *P* 0.34).

All heritability scores, chi-squares and intercepts satisfied the suggested conditions to provide reliable estimates (Supplementary Table 2, available as Supplementary data at *IJE* online). In a positive control analysis, despite lower statistical power (due to the use of two small GWASs: nsCL/P *n* = 5951; philtrum width *n* = 6136), we found suggestive evidence of positive genetic correlation between nsCL/P and philtrum width (r_g_ 0.34, 95% CI −0.06 to 0.73, *P* 0.1) (Supplementary Table 2, available as Supplementary data at *IJE* online). Together, these investigations suggest that our main findings are unlikely to be biased by the small sample size of the nsCL/P GWAS.

### MR

**Figure 2.**
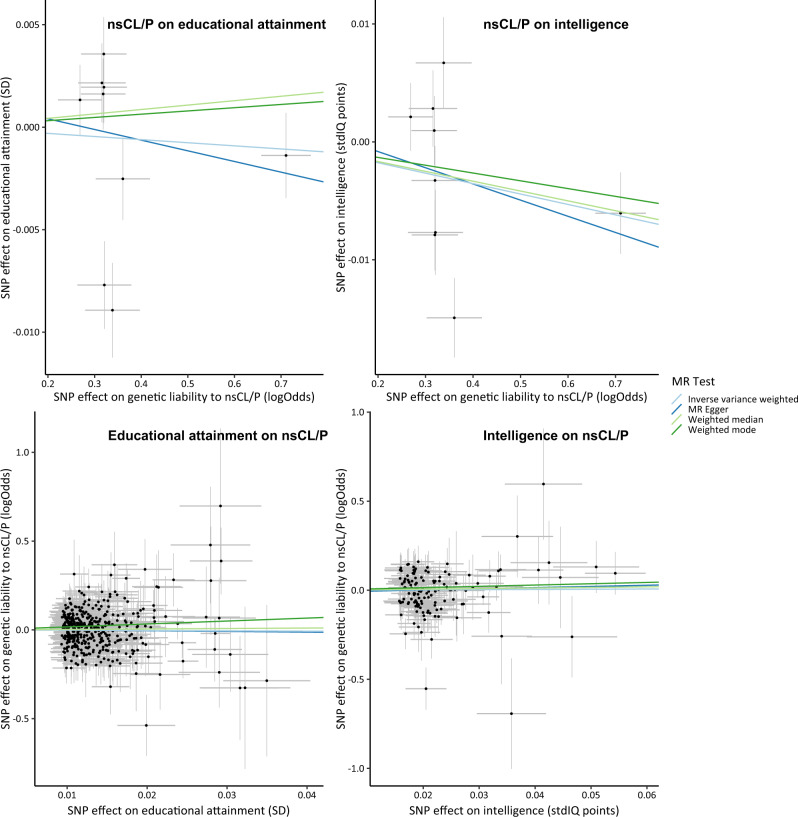
Bidirectional two-sample Mendelian randomization results for associations between genetic liability to nsCL/P, educational attainment and intelligence, using four sensitivity analyses (inverse variance weighted, MR Egger, weighted median and weighted mode). SNP, single nucleotide polymorphism; SD, standard deviation; stdIQ, standardized IQ; nsCL/P, non-syndromic cleft lip with or without palate.

We assessed the strength of the nine instruments for nsCL/P using the F-statistic. The mean F-statistic of the instruments was 53.5, suggesting adequate strength. Using bidirectional two-sample MR, we found little evidence to suggest that genetic liability to nsCL/P influences educational attainment (IVW estimate −0.002; 95% CI −0.009 to 0.006; *P* 0.679). Although the MR estimate implies that a doubling in the genetic liability to nsCL/P decreases years of education by 0.004 years or around 1.6 days, the CI crosses the null (−0.025 to 0.017 years of education per doubling in the genetic liability to nsCL/P). We also found little evidence for an effect of genetic liability to nsCL/P on intelligence (IVW estimate −0.009; 95% CI −0.02 to 0.002; *P* 0.11). The MR estimate implies that a doubling in the genetic liability to nsCL/P decreases intelligence by 0.09 IQ points but, again, the CI crosses the null (−0.2 to 0.02 IQ points per doubling in the genetic liability to nsCL/P). These results were robust to sensitivity analyses using MR Egger, the weighted-median and the weighted-mode approach (Figure 2; Supplementary Tables 4 and 6, available as Supplementary data at *IJE* online). There was little evidence of horizontal pleiotropy bias in the causal estimate, as indicated by the MR Egger intercept (for educational attainment: 0.001, *P* 0.76; for intelligence: 0.002, *P* 0.79). Repeating our analysis using the 12 SNPs found to be strongly associated with nsCL/P in a previous nsCL/P GWAS meta-analysis published by Ludwig *et al.*^30^ also did not change our findings (Supplementary Table 7, available as Supplementary data at *IJE* online).

We found little evidence of a causal effect of (parental) educational attainment on liability to nsCL/P (IVW odds ratio 0.96, 95% CI 0.82 to 1.12, *P* 0.58). Similarly, there was little evidence of a causal effect of (parental) intelligence on offspring liability to nsCL/P (IVW odds ratio 1.01, 95% CI 0.96 to 1.05, *P* 0.74) (Figure 2 and Supplementary Tables 9 and 11, available as Supplementary data at *IJE* online).

Instruments for both analyses had adequate strength and therefore the IVW estimate was unlikely to be affected by weak instrument bias (mean F for educational attainment ≈45; mean F for intelligence ≈43).

## Discussion

### Summary of main findings

We found little evidence that educational attainment and intelligence were genetically correlated with, or affected by, genetic liability to nsCL/P. The large sample sizes in the GWASs of educational attainment and intelligence mean that this study was well powered to detect an effect of nsCL/P, if it exists. Furthermore, it is possible that a small proportion of participants in the outcome GWASs were born with a cleft, which would have biased our estimates away from the null if a strong negative observational correlation between nsCL/P and education or intelligence exists, because this would induce a correlation between high genetic liability to cleft and lower education. Our null results therefore imply that individuals with a high genetic liability to nsCL/P are unlikely to be genetically predisposed to spend less time in education or have lower intelligence (Explanation A in Figure 1). It seems more likely that the observed associations between nsCL/P and low educational attainment are explained by downstream, mediating factors related to being born with a cleft, i.e. expressing the cleft phenotype (such as time spent under anaesthesia, experience of bullying, impaired speech and delayed language development; Explanation B) or environmental confounding factors (such as socio-economic position or parental health behaviours; Explanation C). This finding will help to tailor interventions and policies that target factors influencing the observed associations to effectively improve educational attainment in this population.

### Comparison to previous evidence

In a previous study,^25^ we found evidence that genetic liability to nsCL/P can influence facial morphology (specifically, philtrum width) in the general population, but the current study suggests it is unlikely that there is a similar relationship for educational attainment or intelligence.

There is evidence from the literature that nsCL/P is associated with downstream factors that might mediate any association between nsCL/P and educational attainment (Explanation B). Children born with a cleft lip with palate are at higher risk of poor speech outcomes at 3 years old (i.e. before entering school) and persistent speech disorder,^23^ both of which are strongly associated with lower educational attainment.^44^ Teasing and bullying by peers is common in children born with cleft lip with or without palate,^45^ which can affect psychological wellbeing, enjoyment of school and attainment.^46^ There is also some evidence that teachers perceive the behaviour and abilities of children born with a cleft differently from their classmates.^47^ Affected children are required to take time off school to undergo surgery to repair the cleft (a study in the USA showed that ∼24% of surgeries to repair CL and 37% of surgeries to repair CP are secondary surgeries, and ∼70% of those occur during school ages^48^) and to attend follow-up health assessments, which could affect their learning. There is some observational evidence that repeated surgery (and therefore repeated exposure to general anaesthesia) is associated with lower IQ in children born with a cleft.^20^^,^^49^

There is also evidence suggesting that observed associations between nsCL/P and educational attainment might be explained by confounding (Explanation C). A registry-based study found similar levels of academic achievement in children with nsCL/P and their unaffected siblings,^14^ which could indicate that any attainment deficit in children with nsCL/P is related to features of the family environment that are shared by unaffected family members. An alternative explanation for this finding is that the unaffected sibling is treated differently from the affected sibling in a way that reduces their educational attainment, e.g. through divergence of parental attention and resources to the affected sibling.

Parental health behaviours, such as maternal smoking or alcohol consumption during pregnancy, have been linked to higher rates of nsCL/P^50^ and lower IQ and educational attainment in the general population.^51^^,^^52^ In addition, many of the suggested risk factors for both nsCL/P and low educational attainment might be explained by confounding by lower family socio-economic position, which has also been associated with nsCL/P.^53^ In this study, we found little evidence for a causal effect of parental educational attainment on offspring nsCL/P. This does not support the hypothesis that familial socio-economic position is a causal risk factor for nsCL/P. However, it should be noted that this interpretation is based on the assumptions that (i) years of schooling are good indications of socio-economic position, (ii) genetic variants in offspring are suitable instruments for parental educational attainment and (iii) the analysis was adequately powered to detect a clinically meaningful increase in risk.

### Strengths and limitations

The strengths of this study include: the novel application of a causal inference method (namely MR) to the effects of genetic liability to nsCL/P on education and intelligence; the use of non-overlapping samples drawn from the same population (European descent); the large sample sizes of the educational attainment and intelligence GWASs and statistical power of the MR analyses to detect small effects of genetic liability to nsCL/P on these outcomes; the use of sensitivity analyses to test the robustness of our findings; and the publication of all the data and code used to conduct our analysis, which we hope will facilitate reproducibility and foster a culture of open science in cleft research.

There are also several factors that limit the interpretation of our findings: first, although we used the largest nsCL/P GWAS data set that was available at the time, it was relatively small (*n* = 5951) and LD-score regression can provide unreliable estimates with small GWASs.^37^ However, by showing a correlation between genetic liability to nsCL/P and philtrum width (as a positive control), and by fulfilling several conditions of reliable estimation as set out by the developers of the approach, we provided evidence that our findings are unlikely to be biased by the small sample size of the nsCL/P GWAS. In addition, we found little evidence of an effect of education on liability to nsCL/P. This could be because our estimates were not precise enough to detect the true causal effect. With only 5951 samples (1692 nsCL/P cases), this analysis had low power to detect modest effects.

Second, MR has several limitations (discussed in detail elsewhere^28^^,^^54^^,^^55^) such as unbalanced horizontal pleiotropy, which would violate one of the MR assumptions (when an SNP influences the outcome through a pathway other than via the exposure). We investigated this possibility using multiple independent genetic instruments as a sensitivity analysis (MR Egger, the weighted-median and the weighted-mode approach). We found little evidence of pleiotropy. Furthermore, horizontal pleiotropy typically induces false-positive findings, but is less likely to cause false-negative results. Another limitation is the potential for population stratification (i.e. the different distribution of SNPs across populations of different ancestry) to introduce bias in estimates.^56^ However, we obtained comparable results in our primary analyses and our analyses using SNPs identified in individuals of European descent only, which suggests that our results are unlikely to be confounded by population stratification.

Third, due to the design of the initial nsCL/P GWAS, which combined cleft lip only (CLO) with cleft lip with palate (CLP), we were unable to study subtype-specific effects, including any effect of the cleft palate only (CPO), which was not studied in the GWAS we used. The rationale for excluding CPO cases is that strong prior evidence indicates that nsCL/P and CPO are aetiologically distinct with minimal evidence for genetic overlap.^57^^,^^58^ Furthermore, the sample size for a GWAS of CPO would provide insufficient power for the approaches used in this paper. Findings from previous observational studies suggest that the orofacial cleft subtype is a strong predictor of academic outcomes.^13^ Specifically, children with CPO are at higher risk of underperforming in several areas of academic learning compared with both their unaffected peers and also children born with CLO or CLP.^59^ On the contrary, children born with CLO have been found to have academic achievement higher than children born with CLP or CPO^15^ and sometimes^60^ (though not always^13^^,^^15^) in line with children born without a cleft. There is also some evidence that educational attainment might differ according to the side of the face affected by a cleft^61^ but information on laterality was not available for these GWASs.

Finally, because GWASs typically focus on common genetic variants, we were not able to investigate the potential contribution of rare genetic variants in explaining any shared genetic aetiology between nsCL/P, educational attainment and intelligence. High SNP heritability and low familial recurrence rates suggest that a substantial proportion of genetic liability to nsCL/P is likely to be captured by common genetic variation, but whole-exome sequencing studies suggest that rare variants also contribute to the genetic aetiology of nsCL/P.^62^^,^^63^ Furthermore, rare variants may cause syndromes involving CL/P, which could be misclassified as non-syndromic if the syndromes are difficult to identify clinically.

### Future work

This study highlights the need for further research to understand the multiple potential causes of lower educational attainment in individuals born with any type of orofacial cleft.

There is evidence suggesting associations between nsCL/P and specific cognitive abilities.^64–66^ Although our results suggest that any effect of nsCL/P on specific cognitive abilities is unlikely to influence overall intelligence or educational attainment, further research into these observed associations could provide important information towards developing specialized educational intervention programmes.

In addition, historical educational reforms in Sweden (extending compulsory education from 7 to 9 years) and the UK (e.g. raising the school leaving age to 16 years old in 1972) and other countries could offer the opportunity to investigate the effect of parental educational attainment on risk of nsCL/P and low educational attainment in the offspring. Such research would ideally require population data on the incidence of nsCL/P by month of birth.

There is an increasing need for large-scale, longitudinal data on children born with a cleft and their families, combining genetic data with detailed information on demographic, clinical, psychosocial, environmental and developmental factors. The Cleft Collective Cohort Study^67^^,^^68^ was established in 2013 to address this need and help identify predictive and causal risk factors for cleft and cleft-related outcomes, including educational attainment. Its aim is to enable development of better strategies to facilitate early intervention to improve suboptimal outcomes in individuals born with a cleft. The Cleft Collective welcomes and encourages researchers to apply to use this valuable data resource.

## Conclusion

This study shows that common genetic variants are unlikely to predispose individuals born with nsCL/P to low intelligence or educational attainment. This is an important step towards understanding the underlying aetiology of low educational attainment in this group. The finding is expected to impact family counselling and coping strategies. It might impact the way in which people born with cleft view and define themselves, as well as public perceptions of them. Our findings also encourage further research into the possible common causes of cleft and low educational attainment, and the contribution of downstream factors related to having a cleft. In the current absence of any targeted educational interventions or supportive policies for individuals born with a cleft and their parents, such research will be an important step towards improving educational outcomes in this group.

## Funding

The Medical Research Council (MRC) and the University of Bristol support the MRC Integrative Epidemiology Unit (MC_UU_12013/1, MC_UU_12013/9, MC_UU_00011/1, MC_UU_00011/5). The Scar Free Foundation supports the Cleft Collective (REC approval 13/SW/0064). The Economics and Social Research Council (ESRC) support NMD via a Future Research Leaders grant (ES/N000757/1). C.D. is funded by the Wellcome Trust (108902/B/15/Z). The POFC cohort collection effort and University of Pittsburgh authors are supported by the following grants from the National Institute of Dental and Craniofacial Research (NIDCR): R01-DE016148, X01-HG00784, R01-DE016930, R21-DE012472, R01-DE011931. GCS’s contribution to this work is supported by the Medical Research Council (New Investigator Research Grant, MR/S009310/1) and the European Joint Programming Initiative ‘A Healthy Diet for a Healthy Life’ (JPI HDHL, NutriPROGRAM project, UK MRC MR/S036520/1). No funding body has influenced data collection, analysis or its interpretation.

## Supplementary Material

dyaa047_Supplementary_Data
